# Dynamical Analysis and Visualization of Tornadoes Time Series

**DOI:** 10.1371/journal.pone.0120260

**Published:** 2015-03-19

**Authors:** António M. Lopes, J.A. Tenreiro Machado

**Affiliations:** 1 Institute of Mechanical Engineering, Faculty of Engineering, University of Porto, Porto, Portugal; 2 Institute of Engineering, Polytechnic of Porto, Porto, Portugal; National Scientific and Technical Research Council (CONICET)., ARGENTINA

## Abstract

In this paper we analyze the behavior of tornado time-series in the U.S. from the perspective of dynamical systems. A tornado is a violently rotating column of air extending from a cumulonimbus cloud down to the ground. Such phenomena reveal features that are well described by power law functions and unveil characteristics found in systems with long range memory effects. Tornado time series are viewed as the output of a complex system and are interpreted as a manifestation of its dynamics. Tornadoes are modeled as sequences of Dirac impulses with amplitude proportional to the events size. First, a collection of time series involving 64 years is analyzed in the frequency domain by means of the Fourier transform. The amplitude spectra are approximated by power law functions and their parameters are read as an underlying signature of the system dynamics. Second, it is adopted the concept of circular time and the collective behavior of tornadoes analyzed. Clustering techniques are then adopted to identify and visualize the emerging patterns.

## Introduction

A tornado is a violently rotating column of air extending from a cumulonimbus cloud to the ground. Tornadoes may assume different shapes and sizes and, typically, appear as a funnel with the narrower end touching the ground. Tornadoes’ damaging paths can surpass 1.5 km wide and 80 km long and the most violent events can cause huge destruction and fatalities [1]. Tornadoes are classified according to their intensity and damaging effects [2–3]. Earlier events use the Fujita scale [4–6], while most recent tornadoes are often classified using the enhanced Fujita scale, which was adopted in the United States on February 1, 2007 [7]. In both classification systems, the least damaging tornadoes are rated as F0, while the most damaging ones are rated as F5.

Often, Fujita scale relates the damage caused by a tornado to wind speed classes, according to the empirical equation [8]:

$$v(F)=6.3{\left(F+2\right)}^{3/2}$$ (1)

where *v* represents wind velocity and *F* is the Fujita scale value.

Pursuing a clear physical meaning, other indices have been used to quantify tornadoes intensity, namely the maximum horizontal wind speed, the maximum kinetic energy and the maximum energy flux density [8–10]. Schielicke and Névir [11] introduced the concept of atmospheric moment, *M*_*a*_, analogous to the seismic moment that describes the strength of earthquakes. The value of *M*_*a*_ may be estimated by:

$$M_{a}\approx AL\bar{\rho }e$$ (2)

where, *A* = π*W* ^2^/4 is the circular area of the tornado on the ground, *W* and *L* denote the tornado width and path length, respectively, $\bar{\rho }$ represents the average air density (which is assumed to be equal to 1 kg/m^3^) and *e* is the mass-specific kinetic energy, given by:

$$e=\frac{{\langle v(F)\rangle }^{2}}{2}$$ (3a)

$$\langle v(F)\rangle =6.3{\left(F+2.5\right)}^{3/2}$$ (3b)

Where ⟨*v*(*F*)⟩ denotes the average value of each wind velocity class, obtained by adding the constant 0.5 to each value of *F*.

Expression (2) implicitly assumes a constant tornado intensity and width, whereas those properties often change considerably during the tornado lifecycle. Despite this drawback, the atmospheric moment, *M*_*a*_, has the advantage of being analogous to the seismic moment that describes the strength of earthquakes [11]. Such analogy allows comparing tornadoes and earthquakes, which have been widely studied in terms of their space-time statistical distributions [12].

Tornadoes reveal high variability in terms of intensity, geometric properties (e.g., width and path length) and temporal behavior [12], which is an indicator of the existence of fat tail distributions. In fact, tornadoes have features that are well described by power law (PL) functions [11–14] and unveil characteristics that are also found in complex systems [15–17]. This is the case of Earth’s atmosphere, which is a typical example of an open complex dissipative system, with external forcing caused by the differential heating of the solar radiation [11]. While there are no definitive conclusions about the physical significance of PL, some mechanisms generating such distributions are consistent with self-organized criticality (SOC) [18] and highly optimized tolerance (HOT) [19]. SOC is a process in which a system, by itself, converges to an ordered state, characterized by the emergence of a coherent global pattern created by interactions between low-level entities. The HOT conceptual framework explains the formation of scale-invariant patterns in complex systems. It emphasizes that PL behavior results from optimization of tradeoffs between system yield and tolerance to risk, which drives the system to a specific configuration. Other PL generative mechanisms are addressed by Newman [20].

Tornadoes have been investigated from complementary perspectives and using several statistical tools [8, 10, 21–22]. In the last years relevant studies have been published about this topic. In reference [22] it is shown that tornadoes intensities, expressed in terms of wind speed or in Fujita scale, can be described by Weibull distributions, for which the exponential distributions remain as special cases. Dotzek et al. [8] propose that tornado intensities, expressed in terms of the squared maximum horizontal wind speed, can be described by exponential distributions. Brooks [23] studies the statistical relationship between tornado path lengths and Fujita scale intensities. Schielicke and Névir [11] show that tornadoes exhibit PL behavior when intensities are measured by the atmospheric moment, *M*_*a*_, given by expressions (2)–(4). In reference [16] severe tornadoes and tornado outbreaks are analyzed for the period 1982–2011. The touchdown path length, *L*, is used to measure tornadoes intensity. The authors demonstrate that there is a strong linear scaling between the number of severe tornadoes and their total path length in that same year. Moreover, the noncumulative frequency-length statistics of severe tornado path lengths (i.e., 20 < *L* < 200 km) is well approximated by an inverse PL function with exponent approximately equal to 3. Schielicke and Névir [12] compare the statistics of earthquakes and tornadoes, both in terms of intensity and temporal behavior. The results demonstrate that the statistics of tornadoes reveal PL behavior when the temporal resolution is high (i.e., between 10 and 60 min). Furthermore, it is shown that the distributions support the observation that tornadoes form clusters in time. Doswell et al. [24] show that tornado outbreaks are characterized by spatio-temporal clustering, meaning that long memory is present as an underlying feature. Moreover, the authors claim that the total path length of severe tornadoes in a convective day, *L*_*D*_, is the preferred index to quantify the strength of a tornado outbreak. Verbout et al. [25] discuss the number of tornadoes above a given threshold in a convective day as a measure of the strength of a tornado outbreak.

In this paper we study the collective behavior of tornadoes from the perspective of dynamical systems. A public domain database containing the events occurred in the U.S. during the period 1950 up to 2013 is tackled. We model the occurrences as time series of Dirac impulses with amplitude proportional to the index quantifying the size of the events. In this perspective, we are considering the long term dynamics instead of modelling each individual event. The time series are viewed as the output of a dynamical system and, consequently, the data is interpreted as a manifestation of the system dynamics. First, we analyze the annual time series in the frequency domain, by means of the Fourier transform (FT). The amplitude spectra are approximated by PL functions and the parameters are viewed as a signature of the system characteristics. Second, we adopt the concept of circular time and we compute the circular correlation between the annual time series. Based on the maximum correlation and the corresponding time delay, we use clustering and visualization techniques to unveil patterns.

Bearing these ideas in mind, the paper is organized as follows. In section 2 we describe the data tackled in this work. In section 3 we process the tornadoes annual time series in the frequency domain, by means of the FT, and we interpreted the results according to the characteristics of the spectra. In section 4 we correlate the annual time series and we propose clustering and visualization tools to capture hidden patterns. Finally, in section 5, we outline the main conclusions.

## Dataset

We use the U.S. tornado database compiled by the National Oceanic and Atmospheric administration (NOAA), National Weather Service, Storm Prediction Center. Data is available online at http://www.spc.noaa.gov/, containing all reported U.S. tornadoes from year 1950 up to 2013. There are more than 58,000 tornadoes currently in the database. Each event includes, among other features, information about date and time (with one-minute time resolution), Fujita scale, total number of fatalities and injuries, geographic location (i.e., touchdown and liftoff latitude and longitude coordinates), tornado width and path length.

**Fig 1:**
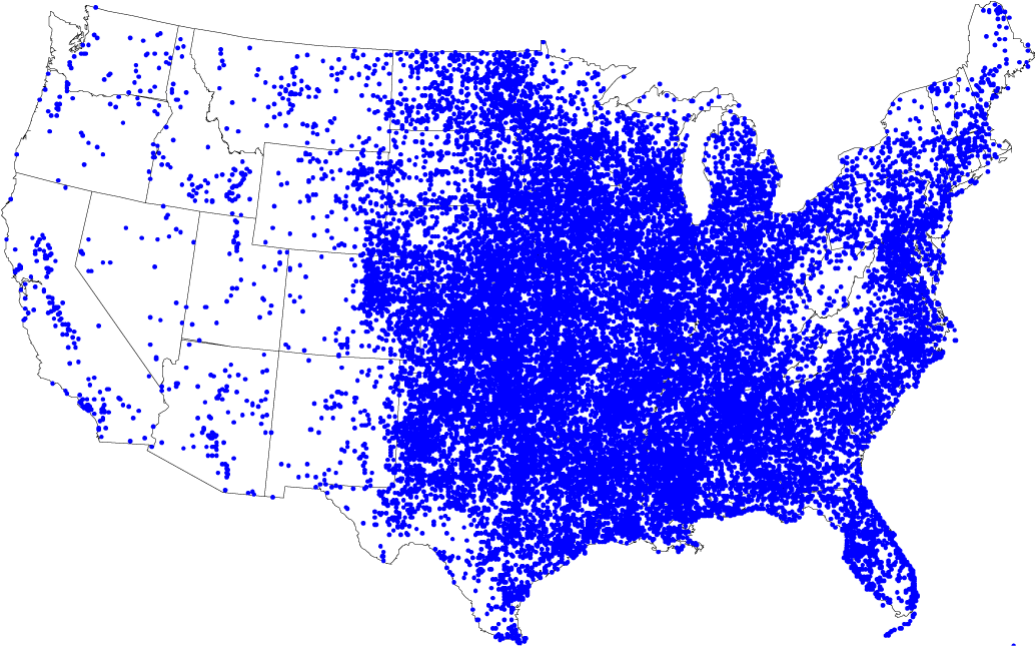
Geographic location of all reported tornadoes that occurred in the U.S., during the time period 1950–2013.

Fig. 1 depicts the geographic location of the events occurred in the U.S., during the time period covered by the database. It can be seen that the East of the Rocky Mountains is particularly prone to tornadoes. This region includes the “Tornado Alley” (i.e., an area centered on Oklahoma, Kansas and northern Texas, and extending from Texas to Canada) and the “Dixie Alley” (i.e., the southern region of the U.S., namely the northern and central parts of Alabama and Mississippi).

**Fig 2:**
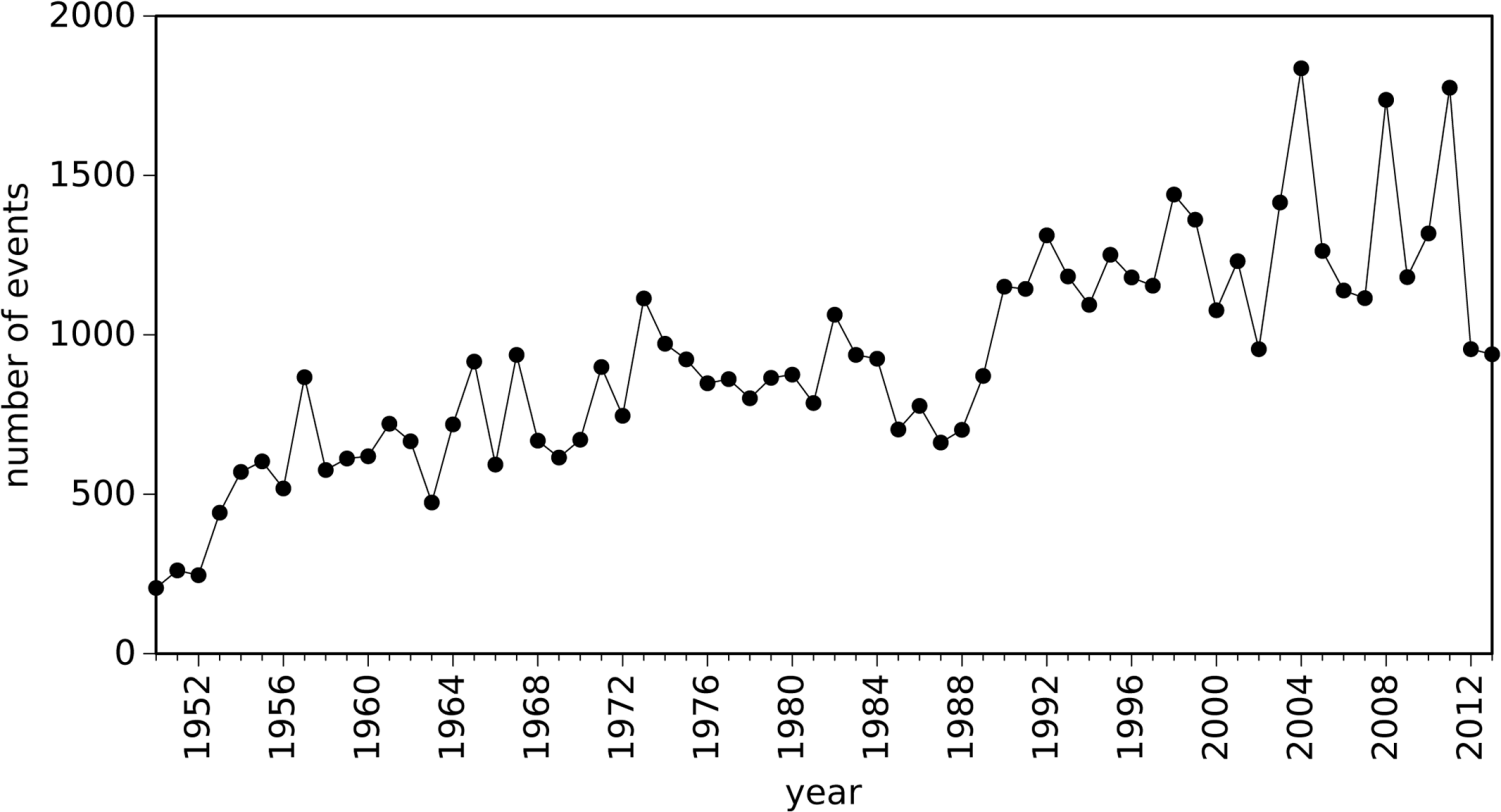
Evolution of the number of reported tornadoes that occurred in the U.S. versus year, during the time period 1950–2013.

Fig. 2 represents the evolution of the number of occurrences versus year, where we can see an increasing trend. However, the statement that we should expect an increasing number of events in the next years is not justified by this simple statistic, as we cannot ignore the fact that there has been a change in the detection capabilities and reporting of the events over time. In fact, in the last decades there has been a more systematic reporting of weak tornadoes over time [21, 23].

**Fig 3:**
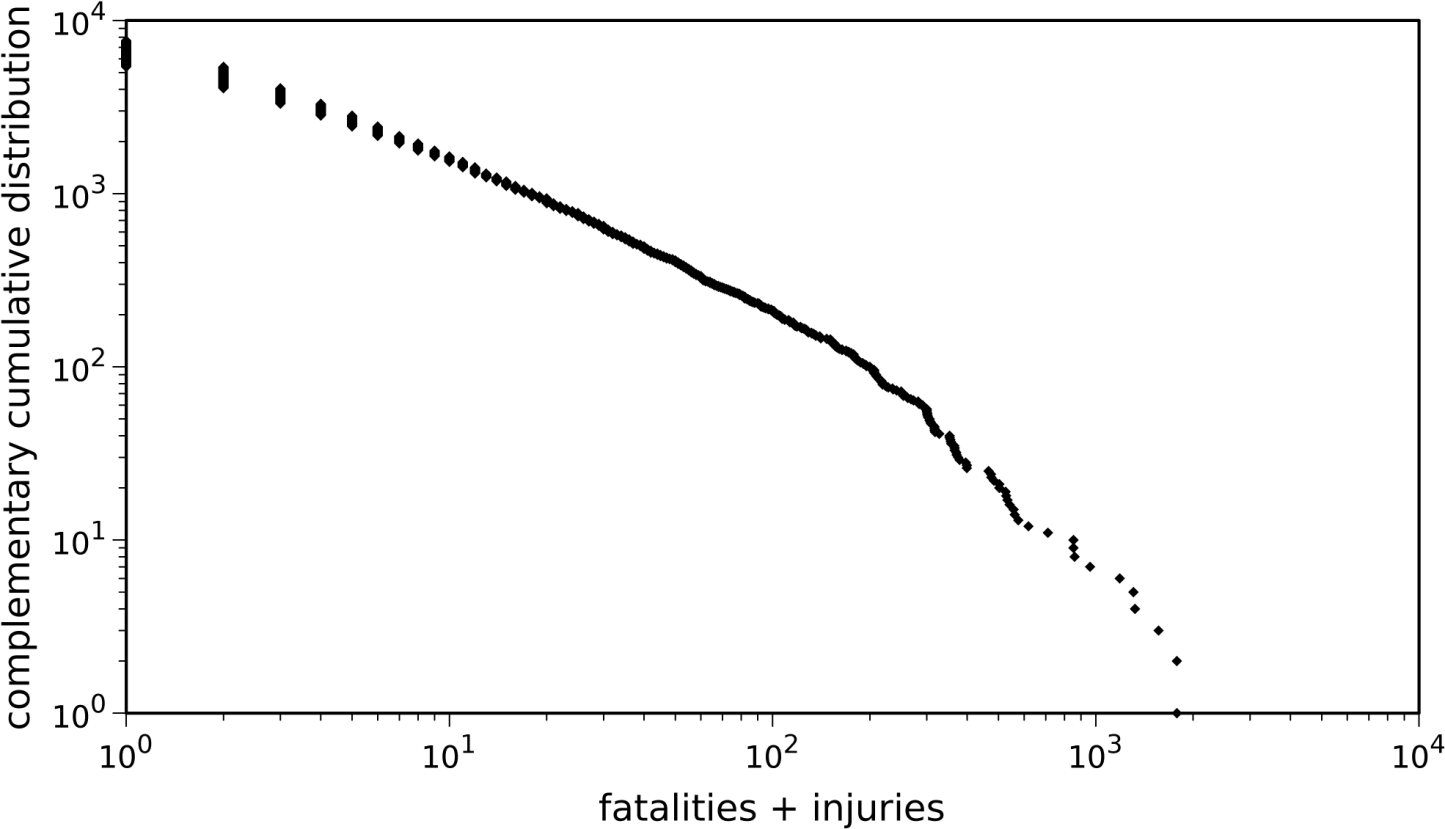
Complementary cumulative distribution of fatalities plus injuries. All reported tornadoes that occurred in the U.S., during the time period 1950–2013 are considered.

The severity of tornadoes, quantified by the total number of affected people (fatalities plus injuries) is represented in Fig. 3, in terms of the complementary cumulative distribution.

**Fig 4:**
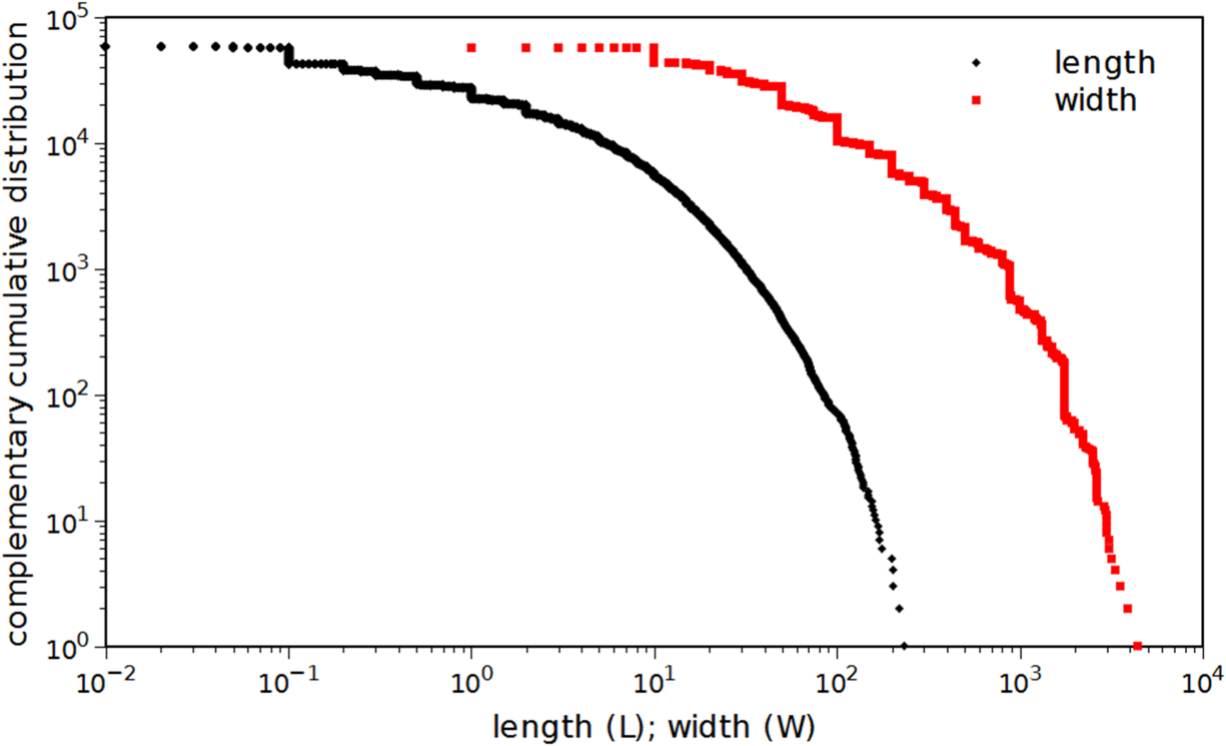
Complementary cumulative distribution of tornadoes path length, *L*, and width, *W*. All reported tornadoes that occurred in the U.S., during the time period 1950–2013 are considered.

Fig. 4 depicts the complementary cumulative distributions of tornadoes path length, *L*, and width, *W*, revealing similar behavior in both cases. It should be noted that the use of fatalities and injuries as a measure of tornado intensity is questionable since it is highly influenced by many factors, namely population density, building codes, safety infrastructure, warning systems and awareness.

These direct statistics reveal limitations for characterizing tornadoes global behavior. In the next sections we adopt several complementary mathematical tools for a deeper understanding of the phenomena. The main tools used, namely FT, hierarchical clustering (HC) and multidimensional scaling (MDS) rely on long time series that are processed in frequency or time domains. FT is a powerful and robust signal processing tool widely adopted in the area of dynamical systems. FT can “dilute” occasional signal artifacts into the final result. HC and MDS have relevant properties for the analysis of complex phenomena since they do not require a priori assumptions on the system nature. Furthermore, since HC and MDS maps do not need data in a uniform grid in time and space, they constitute valuable tools for studying the non-homogeneous data included in tornado catalogues.

## Analysis of Tornado Time Series in the Frequency Domain

In this section we study tornadoes in the perspective of dynamical systems using the Fourier domain. The data is analyzed in an annual basis. For the *i* ^*th*^ case, *i* = 0, …, 63 (cases *i* = 0 and *i* = 63 correspond to years 1950 and 2013, respectively), tornadoes are represented by:

$$x_{i}^{}(t)=\sum_{k=1}^{T}A_{i,k}\delta (t-t_{i,k})$$ (4)

resulting in a total of 64 time series. This means that tornadoes are modelled as Dirac impulses, where *A*_*i*,*k*_ represents the size of the events, measured by an appropriate index, *t*_*i*,*k*_ is the time instant of each occurrence (with one-minute resolution, referred to the beginning of year *i*), *t* represents time, *T* is the period of one year and *δ*(·) corresponds to the Dirac delta function. With this approach, we are not modelling the dynamics of each particular event. Otherwise, we are describing the tornado global dynamics along several decades.

The signal *x*_*i*_(*t*), representative of the collective dynamics of tornadoes, is processed by means of the Fourier transform and the amplitudes of the frequency spectra are approximated by PL functions.

In analytical terms, for the time series signal *x*_*i*_(*t*), we have:

$$F\left\{x_{i}(t)\right\}=X_{i}\left(j\omega \right)=\int_{-\infty }^{+\infty }x_{i}(t)\cdot e^{-j\omega t}\cdot dt$$ (5)

where *F* represents the Fourier operator, *ω* denotes the angular frequency and $j=\sqrt{-1}$.

The PL approximation is given by:

$$\left|F\left\{x_{i}(t)\right\}\right|=\left|X_{i}(j\omega )\right|\approx a_{i}\cdot \omega^{-b_{i}},\;a_{i}\in {ℜ}^{+},\;b_{i}\in ℜ$$ (6)

The values obtained for *b*_*i*_ reveal underlying characteristics of the systems dynamics.

For fitting the model (6) to the experimental data we tried the least squares algorithm, which is known for generating systematic errors under certain conditions [26], the maximum likelihood estimator (MLE) and related statistical tests [26–27] and a genetic algorithm (GA). We obtained identical values for the PL exponents in the three methods. However, as our experimental data is highly “noisy”, the MLE method yields poor statistical significance *p*-values. Therefore, no definitive significance tests are made and no absolute conclusions about PL behavior can be drawn.

We present the results obtained with a standard GA, with elitism, crossover within all population and 5% mutation probability. A population of 5,000 individuals and 1,000 iterations are adopted [28]. For the fitness function we use the Canberra distance between the *N* = 1000 experimental data points, *P* = *p*_1_, *p*_2_,…, *p*_*N*_, and the model, *Q* = *q*_1_, *q*_2_,…, *q*_*N*_:

$$J\left(P,q\right)=\frac{1}{N}\sum_{i=1}^{N}\frac{\left|p_{i}-q_{i}\right|}{\left|p_{i}\right|+\left|q_{i}\right|}$$ (7)

Expression (7) leads to good results since, by calculating the ratio between the difference and the sum of two values, it is possible to capture the relative error of the adjustment, avoiding the undesirable effects that occur when using the standard Euclidean norm due to the simultaneous presence of large and small values.

**Fig 5:**
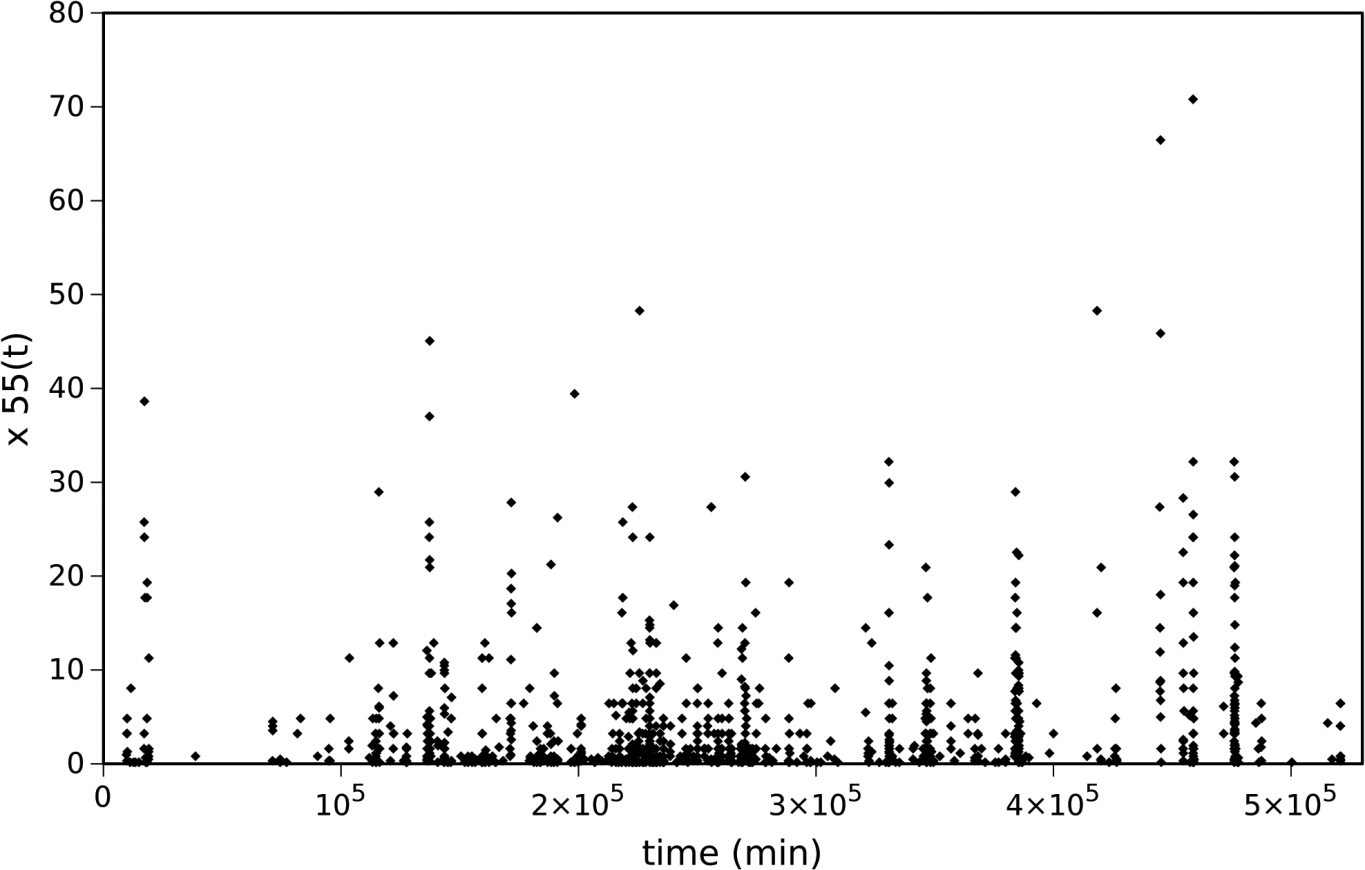
Time series, *x*_55_(*t*), representation of tornadoes path length, *L* (km), in year 2005.

**Fig 6:**
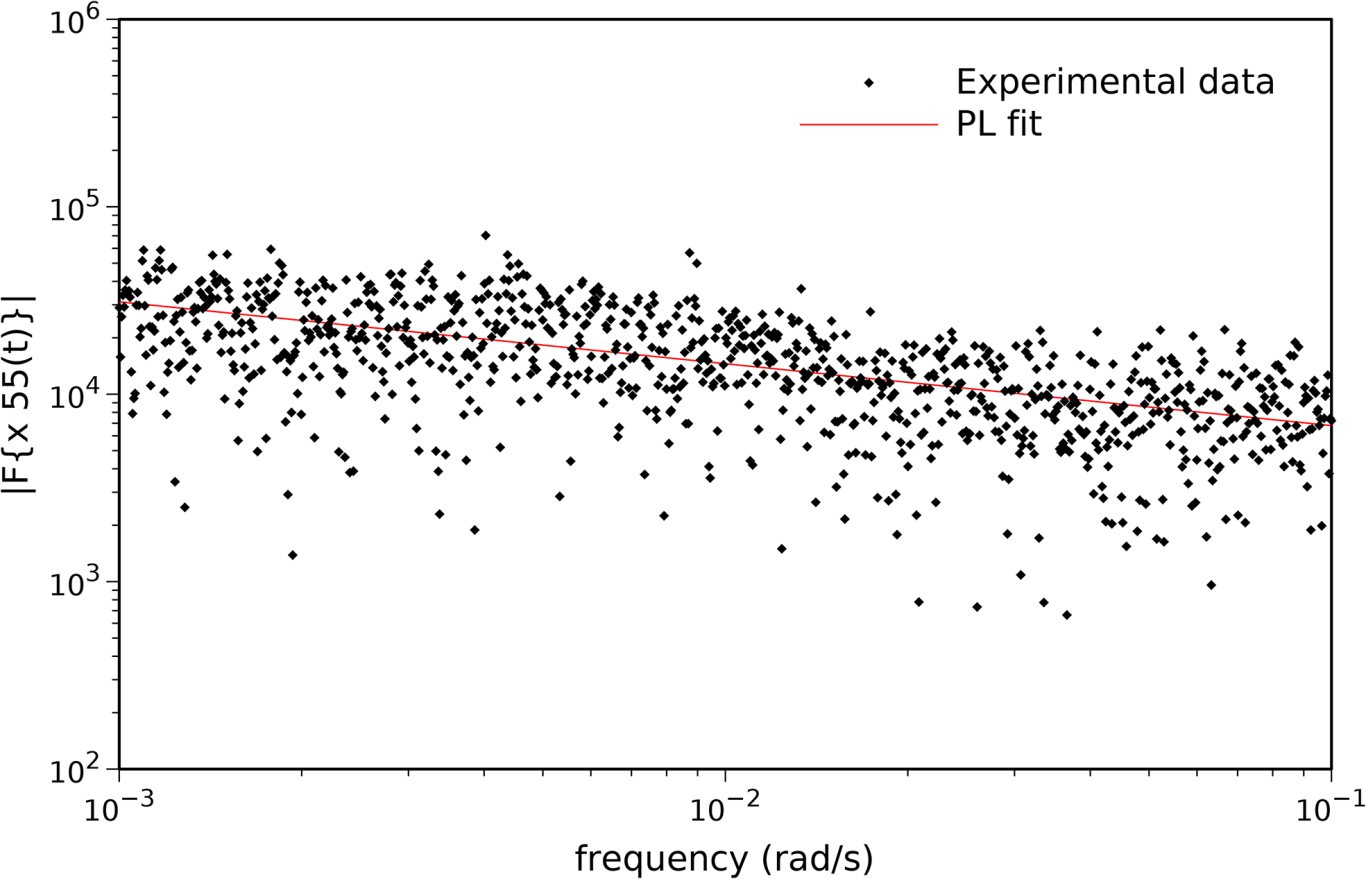
Magnitude of the Fourier transform, |*F*{*x*_55_(*t*)}|, and PL approximation for the time series representation of tornadoes path length, *L*, in year 2005.

Two indices are used to measure tornadoes intensity: tornado path length, *L*, and tornado width, *W*. For example, Fig. 5 depicts the time series of year 2005, *x*_55_(*t*), where amplitude *A*_55_ represents the corresponding tornadoes path length, *L*_55_. Fig. 6 shows the magnitude of the Fourier transform, |*F*{*x*_55_(*t*)}|, and the PL approximation. The resulting parameters are (*a*_55_, *b*_55_) = (3179.7, 0.330), revealing a fractional value of *b*_55_. Alternatively, for *A*_55_ representing tornadoes width, *W*_55_, the results are qualitatively similar, yielding parameters (*a*_55_, *b*_55_) = (149759, 0.280). PL behavior is a characteristic exhibited by long memory systems. Furthermore, Eq. (6) establishes a direct relationship between PL behavior and fractional Brownian motion [29–30], which is a signature of complexity.

**Fig 7:**
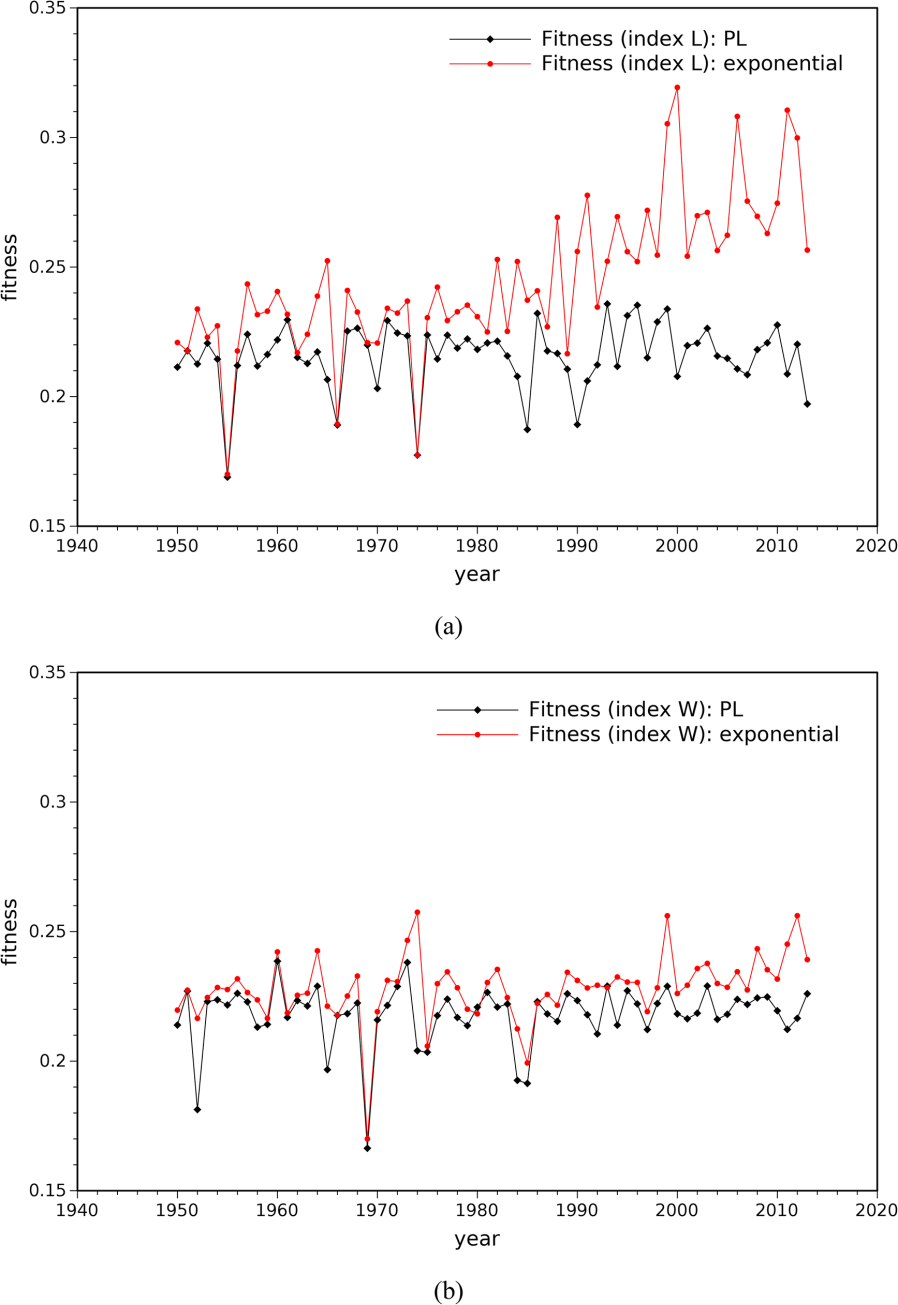
Fitness function values obtained for the PL and the exponential models: (a) tornado path length index, *L*; (b) tornado width index, *W*.

Adopting the procedure described above, the PL parameters are calculated for all years *i* = 0, …, 63. Furthermore, the PL and the exponential approximations are compared. Fig. 7 depicts the fitness function (7) obtained for the PL and the exponential models, both for tornado path length, *L*, and tornado width, *W*, indices. It can be seen that, for all years, the PL model fits well into the experimental data.

**Fig 8:**
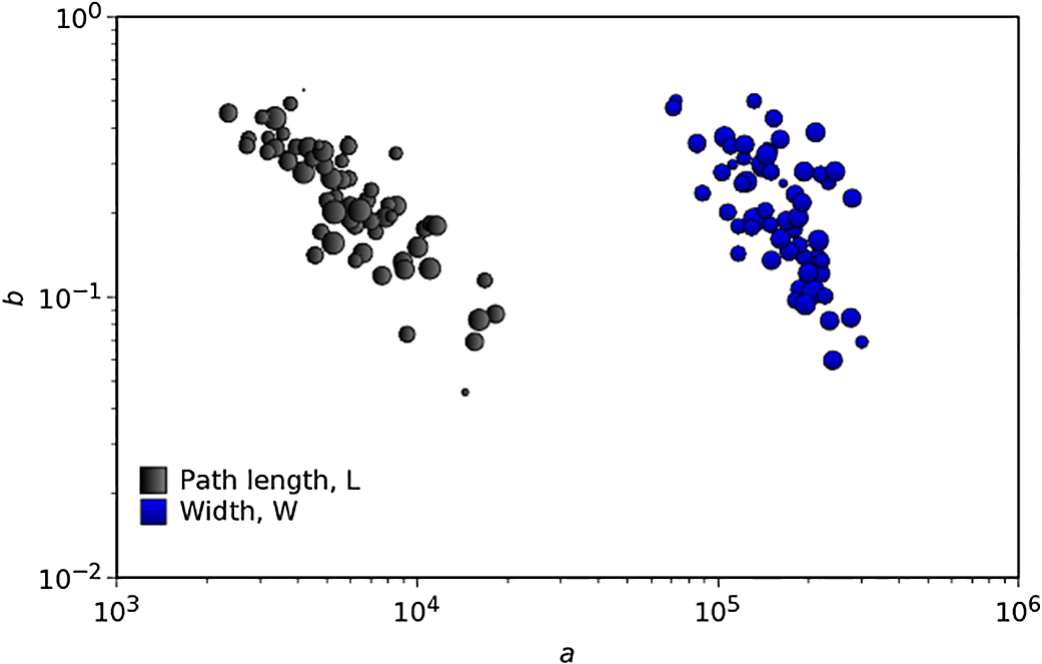
Loci of the PL parameters (*a*_*i*_, *b*_*i*_) for all years, *i* = 0, …, 63, considering the tornadoes path length, *L*, and width, *W*, to quantify intensity. A larger (smaller) diameter of the bubbles means a larger (smaller) value of the fitness function of the PL fit.

Fig. 8 depicts the (*a*_*i*_, *b*_*i*_) loci when considering the tornado path length, *L*, and the tornado width, *W*. A larger (smaller) diameter of the bubbles means a larger (smaller) value of the fitness function of the PL fit. We can observe some kind of “line alignment” regularity in the parameters. The mention to such behavior emerged recently in the scientific literature in phenomena with memory and fractal features, but a definitive justification is still to be found [31–34].

The results show that tornadoes collective behavior exhibit correlations and characteristic patterns. However, complementary analysis is needed to reveal deeper characteristics of the phenomena.

## Analysis of Tornado Time Series in the Time Domain

In this section we analyze the tornado time series, *x*_*i*_(*t*), *i* = 0, …, 63, in the time domain. In subsection 4.1 we introduce de concept of circular time. In subsection 4.2 we compute the circular correlation [35] between the time series and we analyze the temporal behavior exhibited by tornadoes. In subsection 4.3, based on the maximum circular correlation values and the corresponding time delays, we use clustering and visualization tools to unveil patterns.

### The flow of time

The adoption of a non-Cartesian time scale is motivated by the “one-year periodicity” observed in tornado time series, where December is close to January, and not the opposite, as a Cartesian scale implicitly assumes. On the other hand, a given month within one year is expected to be similar (i.e., close in some sense) to the same month in most of other years, or, at least, to the same months in the closest years.

Bearing these ideas in mind, we consider that time evolves along an Archimedean spiral, defined by:

$$\theta =2\pi \cdot \left(\frac{t}{T}+i\right),r=p+q\cdot \theta$$ (8)

where (*r*, *ϴ*) denotes the radius and angle coordinates, respectively, and *i* = 0, …, 63, represents the year. For simplicity, we consider *p* = *q* = 1. We represent the tornadoes by points, where coordinates (*r*, *ϴ*) correspond to the circular (spiral) time of the event. We interpolate the points, associating a color to the intensity, measured by *L* (in log units), and we depict the events on a contour plot. With this representation we preserve the proximities of seasons both within each year and between distinct years. This means, for example, that January of year 2006 is close to December of year 2005, and that January 2005 is close to January 2004 and January 2006.

On the other hand, the major drawback is that initial years (in the center) have a small graphical representation, while recent years (in the outside region) have a larger area.

**Fig 9:**
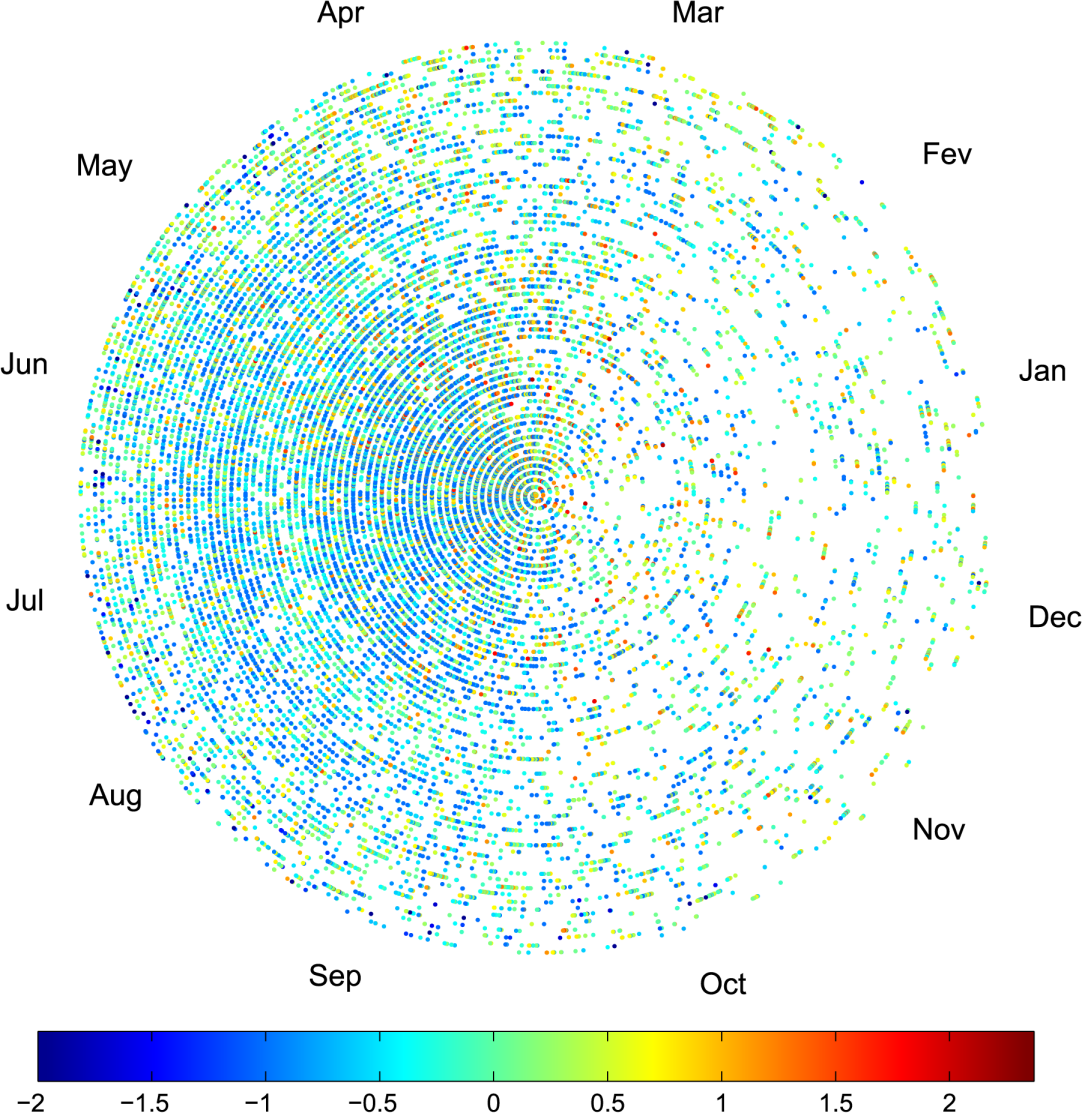
Temporal evolution and intensity (in log units) of the events occurred in the U.S. during the time period 1950–2013. The coordinates (*r*, *ϴ*) represent the circular (spiral) time of the events and the colors represent the intensity, measured by *L* (in log units).

Fig. 9 illustrates the temporal evolution and intensity of the events occurred in the U.S. during the time period 1950–2013. On the left half circle (i.e., for the months from April up to September) we can see many small (in time) events, while, on the right half circle, the events are larger. Higher intensity events are observed in the fourth and first trimester, in that order.

Since the results shown in Fig. 9 close resemble those of chaotic systems, we first analyze the chart by means of the fractal dimension. The fractal dimension is a signature of complex systems, composed by many parts that interact with each other at different scales in time and space, which is the case of the atmospheric phenomena that trigger tornadoes.

We calculate the fractal dimension of sets of points (in a black and white picture) according with a variable threshold level. First, we divide the whole set of events into *l* = 16 groups, as a function of their intensity level, measured by *L* (in log units). Second, we generate the respective monochrome images corresponding to the temporal evolution of the events (i.e., black and white images, similar to Fig. 9). Third, we adopt the box-counting method for estimating the fractal dimension of the plots [36–37]. The value for *l* establishes a compromise between good resolution and a descriptive number of events in each group.

Given a set *S* in a *n*-dimensional space and considering that *N*_*ε*_ (*S*) is the minimum number of *n*-dimensional cubes of side-length *ε* > 0 needed to cover *S*, if there is a number *d* so that *N*_*ε*_(*S*) ∼ 1/*ε* ^*d*^ as *ε* → 0, then the box counting dimension of *S* is:

$$d=-\underset{\varepsilon \to 0}{\lim}\frac{\ln\left[N_{\varepsilon }(S)\right]}{\ln(\varepsilon )},\;\varepsilon >0$$ (9)

In our case *S* consists of monochrome images and small values of *ε* are reached by accessing the images at the pixel level.

**Fig 10:**
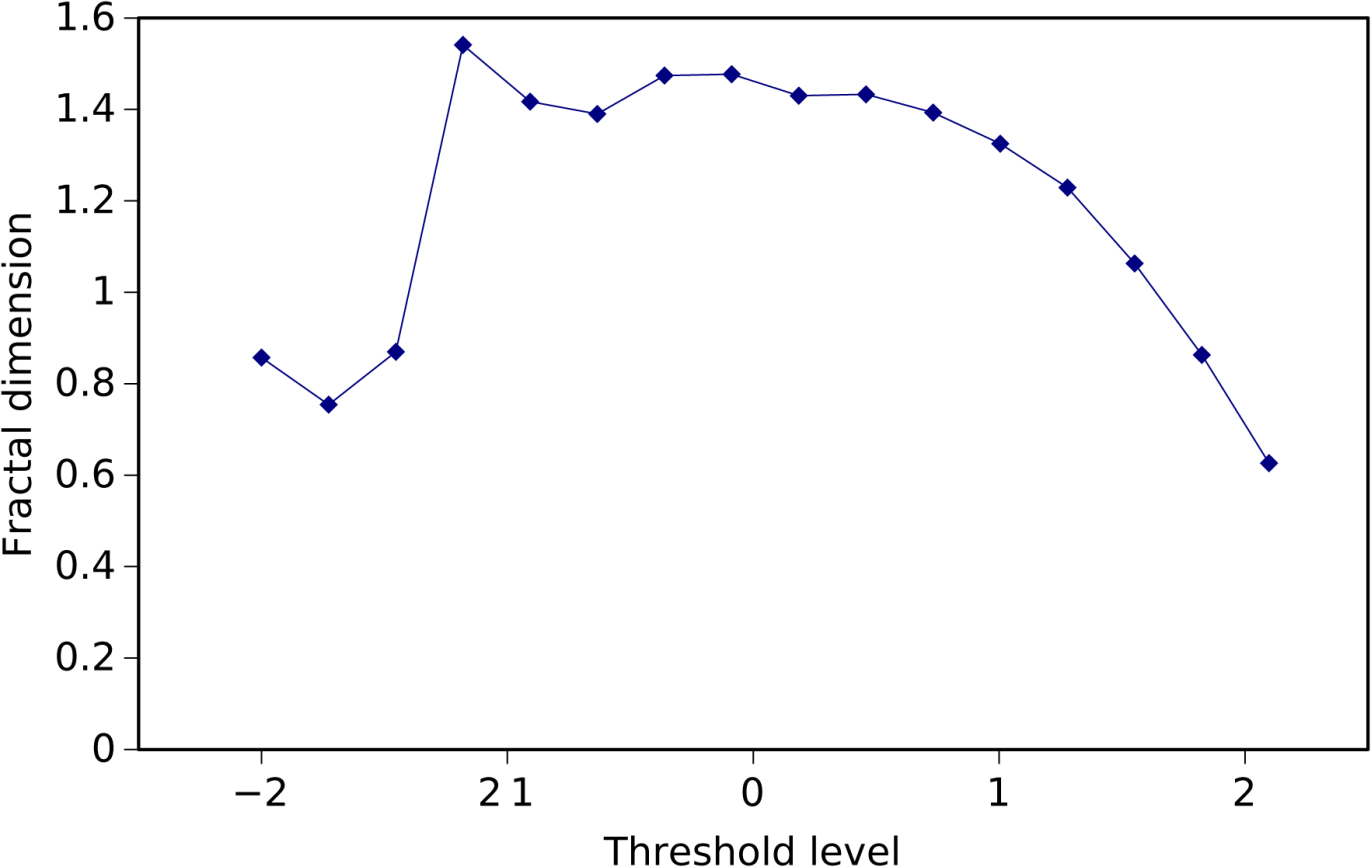
Evolution of the fractal dimension, *d*, for the 16 groups of data obtained according to the intensity level of the events, measured by *L* (in log units). The box counting method is used.

In Fig. 10 we depict *d* for the 16 groups of data. It can be seen that for the first three groups the fractal dimension is small, probably due to missing data corresponding to unreported weak events. For mid-range intensity values *d* is almost constant. The fractal dimension decays for stronger events, since those are less common.

In conclusion, this graphical technique leads to an intuitive representation of data, but does not reveal clear patterns and relationships between sets of data. This means that more advanced processing techniques must be developed.

### Time series correlation

Inspired on the previous sub-section and in the quasi-periodicity of time for the phenomena under study, here we propose analytical techniques based on the concept of “circular time”.

The circular correlation function, *c*_*ij*_(*τ*), compares *x*_*i*_(*t*) with the time delayed signal *x*_*j*_(*t*–*τ*), where time, *t*, is measured with respect to the beginning of the corresponding year. In analytical terms we have:

$$c_{ij}\left(\tau \right)=\frac{\sum_{k=1}^{T}x_{i}(t_{k})\cdot x_{j}(t_{k}-\tau )}{\sqrt{\sum_{k=1}^{T}x_{i}{\left(t_{k}\right)}^{2}\cdot \sum_{k=1}^{T}x_{j}{\left(t_{k}-\tau \right)}^{2}}},\;i,j=0,\cdots ,63$$ (10)

For each case, the delayed signal *x*_*j*_(*t*–*τ*) is treated as a circular buffer. The time delays, denoted as $\tau_{ij}^{*}$, correspond to the time at which the circular correlation has its maximum value, $c_{ij}^{*}=c_{ij}(\tau_{ij}^{*})$.

**Fig 11:**
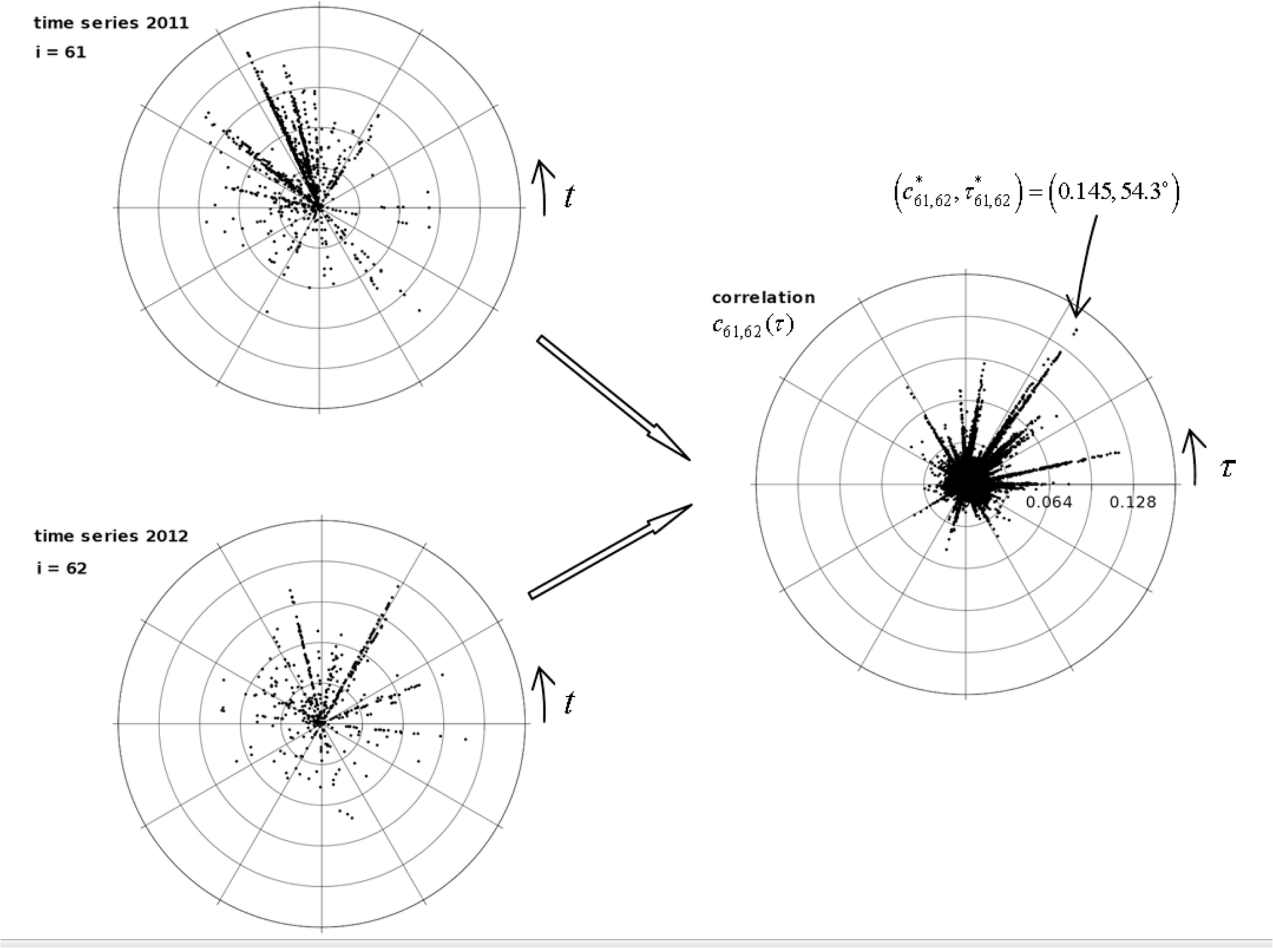
Tornado time series *x*_61_(*t*) and *x*_62_(*t*) (on the left) and correlation function, *c*_*ij*_(*τ*), (on the right) between years 2011 and 2012.

For example, Fig. 11 (on the left part) depicts the time series *x*_61_(*t*) and *x*_62_(*t*) (i.e., years 2011 and 2012), respectively. In both graphs the coordinated (*r*,*θ*) represent intensity (i.e., tornadoes path length, *L*, in logarithmic units) and circular time, respectively. For each time series, time *t* is referred to the beginning of the corresponding year, yielding *θ* = 2*π*·*t*·*T* ^−1^. Fig. 11 (on the right part) shows the correlation function, *c*_61,62_(*τ*). Points (*r*, *ϴ*) correspond to correlation value, *c*_61,62_(*τ*), and (circular) time delay, *τ*, respectively. For this case, we obtain the maximum correlation $c_{61,62}^{*}=0.145$ and time delay $\tau_{61,62}^{*}=79500$ minutes (i.e., 55.2 days, in the time series, or 54.3°, in the polar plot).

**Fig 12:**
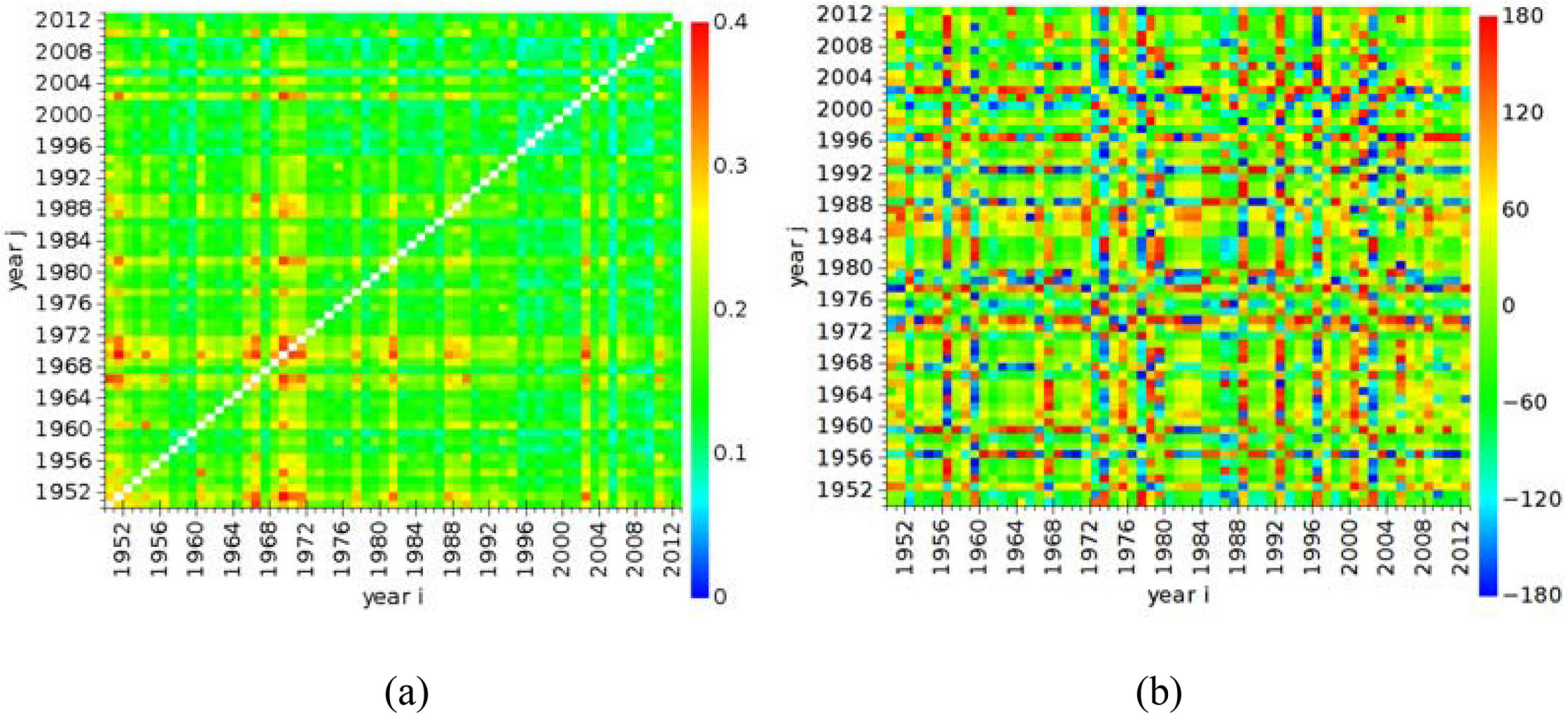
Matrix plots: (a) maximum circular correlation, $c_{ij}^{*}$; (b) time delay, $\tau_{ij}^{*}$, between all pairs of years (*i*, *j*) = 0, …, 63.

We compute the parameters ($c_{ij}^{*}$, $\tau_{ij}^{*}$) for all pairs of years (*i*, *j*) = 0, …, 63, using the procedure described above. The results are illustrated in Fig. 12A-B for, $c_{ij}^{*}$ and $\tau_{ij}^{*}$, respectively. The values for *i* = *j* (i.e., those cases representing autocorrelation) are removed from Fig. 12A in order to avoid saturating the chart.

These maps compare all annual time series in a global perspective. However, the results are difficult to interpret and are most suitable for pairwise comparisons.

### Clustering and pattern visualization

In this subsection, we adopt HC and MDS techniques to reveal and to visualize tornado patterns.

#### Hierarchical clustering

HC is a statistical technique for data analysis [38]. HC aims to build a hierarchy of clusters, in such a way that objects in the same cluster are similar to each other. In agglomerative clustering each object starts in its own singleton cluster and, at each step, the algorithm merges the two most similar (in some sense) clusters. In divisive clustering, all objects start in one single cluster and, at each step, the algorithm removes the “outsiders” from the least cohesive cluster. The results of HC are presented in a dendrogram. Clusters are combined (for agglomerative) or split (for divisive) based on a measure of their dissimilarity. The algorithm uses a metric, to quantify the distance between pairs of objects, and a linkage criterion, to quantify the dissimilarity between clusters. The metric will influence the composition of the clusters and must be chosen carefully. The Euclidean and Manhattan distances and the maximum, minimum and average linkages are often used.

To assess the clustering we use the cophenetic correlation coefficient [39], which is a measure of how well the cluster tree generated by the linkage function preserves the pairwise distances between the original unmodeled data points. If the clustering is valid, the linking of objects in the cluster tree has a strong correlation with the distances between objects in the original data set. The closer the value of the cophenetic correlation coefficient is to 1, the more accurately the clustering reflects the original data. The result is often plotted in a Shepard graph comparing the original and the cophenetic distances. The better the clustering the closer to the 45 degree line the points will lie.

We feed the HC algorithm with the 64×64 matrix $C=\left[\left|\tau_{ij}^{*}\right|\right]$ and we adopt the successive (agglomerative) clustering and average-linkage methods.

**Fig 13:**
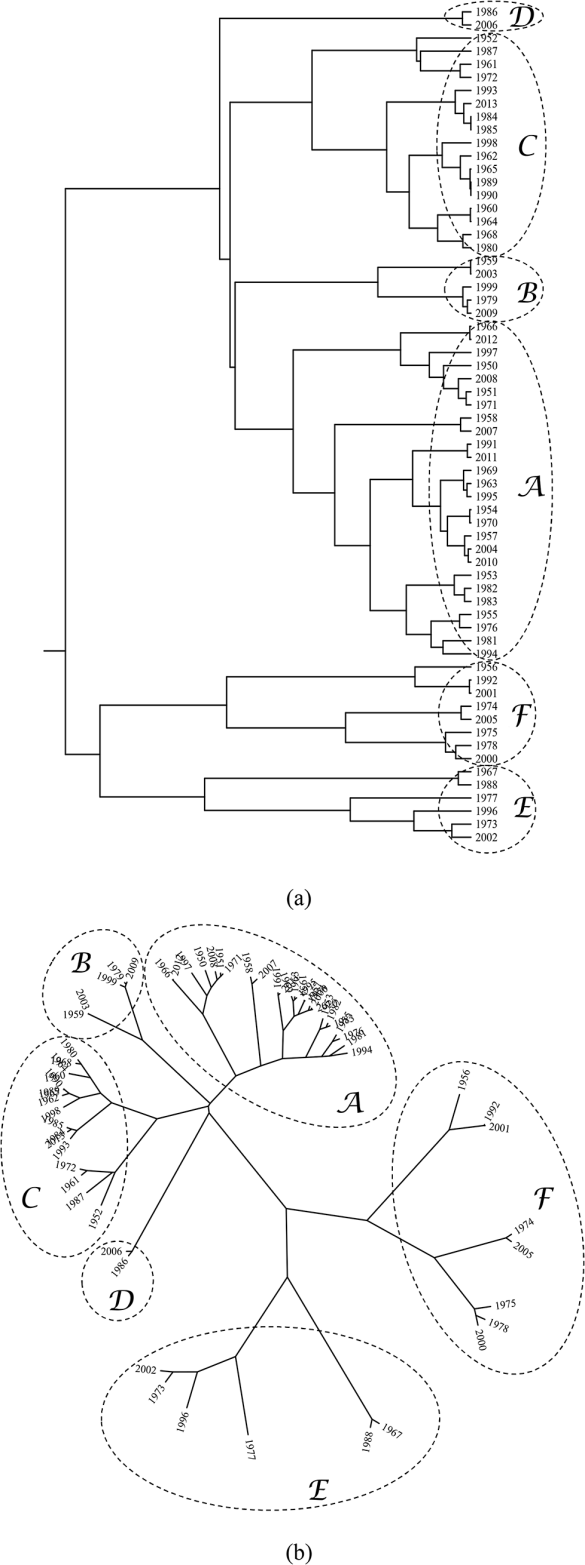
HC graphs: (a) dendrogram; (b) visualization tree. The matrix $C=\left[\left|\tau_{ij}^{*}\right|\right]$ is used.

**Fig 14:**
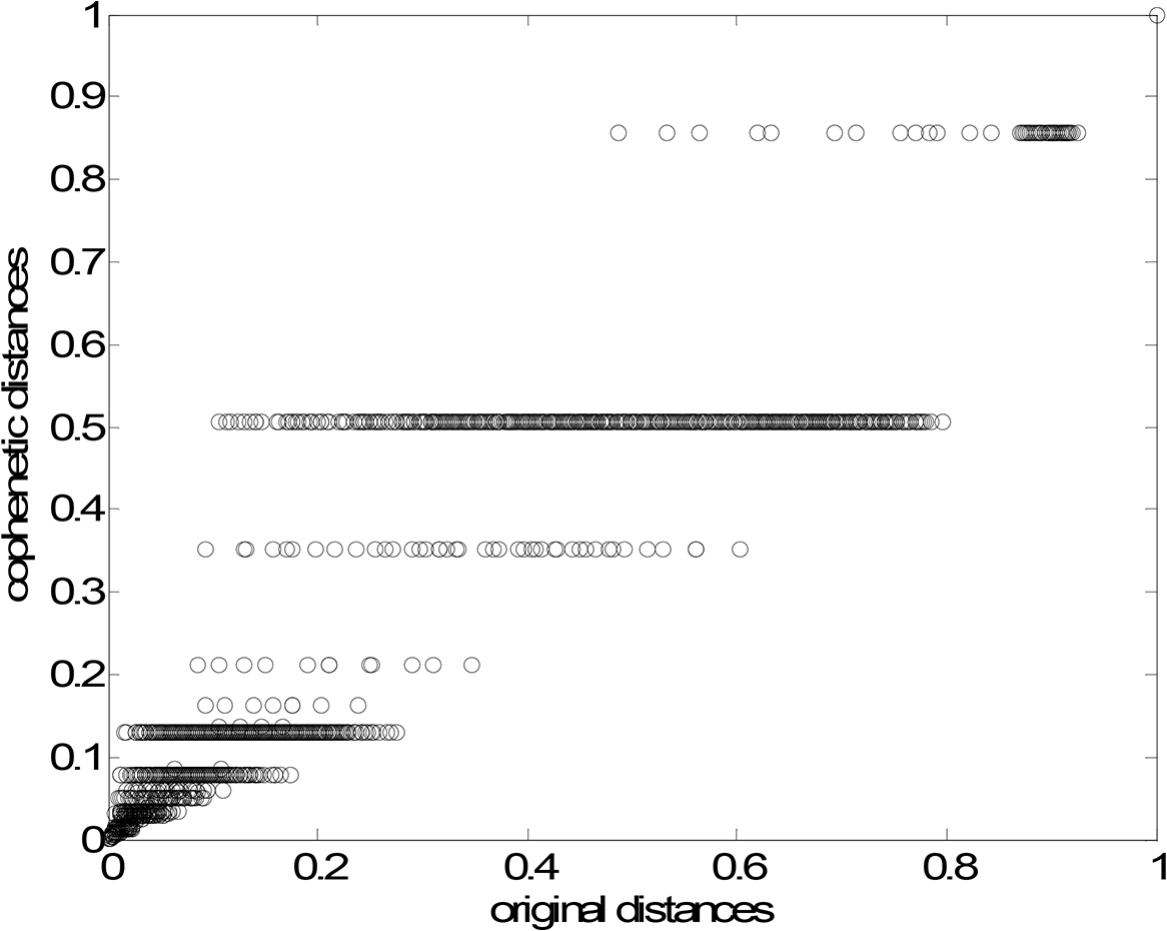
Shepard plot of the HC. The cophenetic correlation coefficient is equal to 0.93.

Fig. 13A and 13B depict the corresponding dendrogram and visualization tree, respectively. As can be seen, two main patterns emerge forming a “dipole”, where each part is composed by the smaller clusters {A, B, C, D} and {E, F}, respectively. Fig. 14 represents the Shepard plot for the HC, reflecting an accurate clustering of the original data, where the cophenetic correlation coefficient is equal to 0.93.

#### Multidimensional scaling

MDS is a statistical technique for visualizing data [40]. MDS generates maps, where objects perceived to be similar to each other are placed in clusters. The algorithm requires a *p* × *p* symmetric matrix, **C**, of item to item similarities (or, alternatively, of similarities), where *p* is the number of objects. MDS assigns a point to each item in a *m*-dimensional space and arranges the objects in order to reproduce the observed similarities (or, alternatively, dissimilarities). This is achieved by evaluating different configurations, while maximizing the goodness-of-fit. The raw stress, *S*, is often used to evaluate how well a particular configuration reproduces matrix **C**:

$$S={\left[d_{ij}-f(\delta_{ij})\right]}^{2}$$ (11)

where, *d*_*ij*_ and *δ*_*ij*_ correspond to the reproduced and to the observed distances, respectively. The function *f*(.) represents a transformation applied to the input data. The smaller the stress, *S*, the better is the fit between the observed and the reproduced data.

For *m* = 2 or *m* = 3 the generated map can be easily interpreted. The actual orientation of axes in the final solution is arbitrary. MDS maps are insensitive to rotations and translations. In fact, MDS maps interpretation is based on the emerging clusters and relative distances, rather than on the absolute coordinates or shapes. The measure for constructing matrix **C** depends of the researcher’s choice.

For accessing the quality of the MDS map there are adopted the stress and Shepard plots. The stress plot, representing *S* versus the number of dimensions *m* of the MDS, leads to a monotonic decreasing chart. Therefore, we choose the “best” dimension *m* as a compromise between stress reduction and number of dimensions for the MDS. The Shepard diagram depicts the reproduced distances, for a particular value *m* of the MDS dimension, versus the observed input data (distances). Therefore, in the Shepard diagram, a narrow scatter around a 45 degree line indicates a good fit of the distances to the dissimilarities, while a large scatter indicates a lack of fit, for that value *m*.

**Fig 15:**
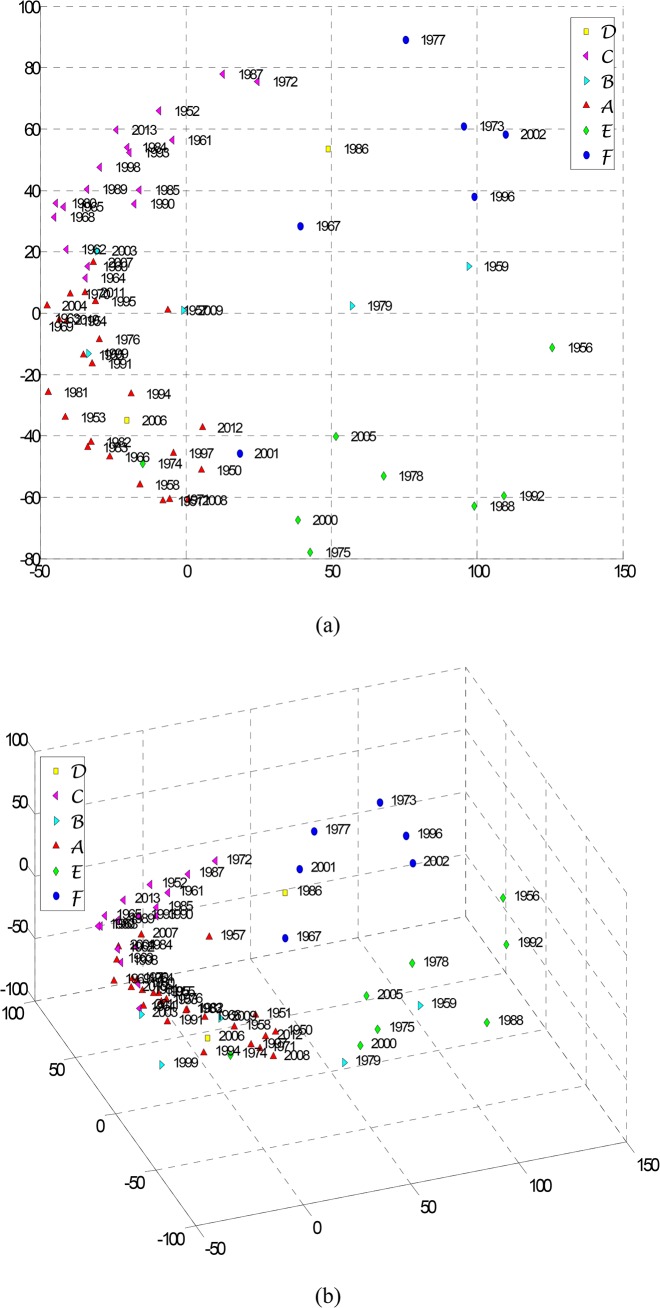
MDS maps: (a) 2-dimensional; (b) 3-dimensional. The matrix $C=\left[\left|\tau_{ij}^{*}\right|\right]$ is used.

**Fig 16:**
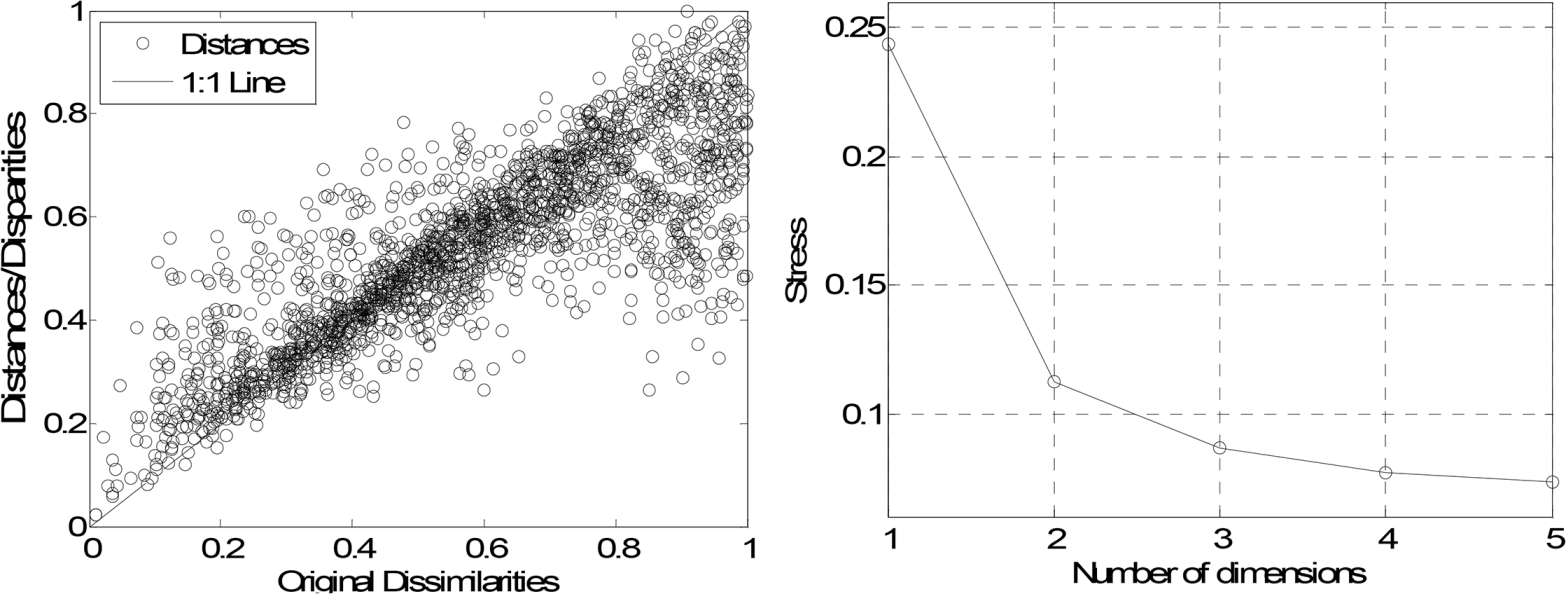
MDS assessment graphs: (a) Shepard map for the 3-dimensional MSD map; (b) stress map.

As before we feed the MDS with the 64×64 matrix $C=\left[\left|\tau_{ij}^{*}\right|\right]$ and generate the 2- and 3-dimensional maps (Fig. 15A and 15B, respectively), where we can identify the “dipole” mentioned previously. To assess the quality of the MDS maps we plot the Shepard and the stress diagrams. The Shepard graph (Fig. 16A) shows the points distributed around the 45 degree line, which means a good fit of the distances to the dissimilarities. The stress plot (Fig. 16B) reveals that a 3-dimensional space describes well the data.

The results obtained for the distinct visualization techniques (Figs. 13 and 15) reveal clusters and, consequently, patterns in time. Nevertheless, the results are far from a simple type of periodicity. This means that either the time series length is insufficient to grab some period, or that some complex long run behavior is present and needs to be further analyzed with complementary techniques. Embedding other variables, namely trajectories, pressures, temperatures and humidity may help getting a clear picture of the global pattern.

## Conclusion

This paper analyzed the collective behavior of tornadoes from the perspective of dynamical systems, due to the fact that such phenomena reveal characteristics found in complex systems. A public domain database of U.S. tornadoes occurred in the time period 1950–2013 was adopted. The events were modeled as time series of Dirac impulses proportional to their intensity. In a first approach, we proposed the Fourier transform was applied to characterize tornadoes behavior. A second approach adopted the concept of circular time and correlation. Hierarchical clustering and multidimensional techniques were used to identify and visualize patterns. It is clear the emergence of clusters corresponding to complex dynamical effects. In fact, the emerging patterns have resemblances with those of chaotic systems that lead to a poor predictability. Furthermore, measuring a richer set of variables and recording of longer time series might be necessary to establish a solid basis of computer data analysis. These approaches confirm that tornado dynamics is complex and exhibits long memory characteristics. The results may inspire still other approaches that, perhaps in combination with those presented, may yield important insights to better understand the phenomenon.
