# Correction: Dynamical Analysis and Visualization of Tornadoes Time Series

**DOI:** 10.1371/journal.pone.0133106

**Published:** 2015-07-14

**Authors:** The PLOS ONE Staff

## Notice of Republication

This article was republished on June 22, 2015, to correct formatting errors in Figures 6, 7, 12, 13, 14, 15 and 16 that were introduced during the typesetting process. The publisher apologizes for the errors. Please download this article again to view the correct version.
